# Impact of Aerogel Modification for Fe−N−C Activity and Stability towards Oxygen Reduction Reaction in Phosphoric Acid Electrolyte

**DOI:** 10.1002/cssc.202401843

**Published:** 2024-12-19

**Authors:** Tanja Zierdt, Torben Reuter, Julia Müller–Hülstede, Julia Buschermöhle, Dana Schonvogel, Jessica Kröner, Marina Schwan, Barbara Milow, Peter Wagner, K. Andreas Friedrich

**Affiliations:** ^1^ Institute of Engineering Thermodynamics German Aerospace Center (DLR) Carl-von-Ossietzky-Str. 15 26129 Oldenburg Germany; ^2^ Institute for Building Energetics Thermotechnology and Energy Storage (IGTE) University of Stuttgart Pfaffenwaldring 31 70569 Stuttgart Germany; ^3^ Institute of Materials Research, Aerogels and Aerogel Composites German Aerospace Center (DLR),Linder Höhe, 51147 Cologne Germany; ^4^ Institute of Chemistry Carl von Ossietzky University Carl-von-Ossietzky-Str. 9–11 26129 Oldenburg Germany; ^5^ Institute of Engineering Thermodynamics German Aerospace Center (DLR) Pfaffenwaldring 38–40, 70569 Stuttgart Germany

**Keywords:** Fe−N−C, Electrocatalyst, Carbon aerogel, Oxygen reduction reaction, Phosphoric acid electrolyte

## Abstract

Resorcinol‐formaldehyde based carbon aerogel (CA) has been tailored to meet the requirements as a Fe−N−C carbon support, aiming to provide sufficient, inexpensive cathode catalysts for high‐temperature polymer electrolyte membrane fuel cells (HT–PEMFCs). Therefore, different treatments of the aerogel are explored for optimal pore structure and incorporation of surface functionalities, which are crucial for Fe−N−C synthesis and electrochemical performance. Fe−N−Cs of differently modified aerogel are investigated in phosphoric acid electrolyte. The results show that HNO_3_ treatment for 5 h yields the Fe−N−C with highest mass activity and selectivity, attributed to the highest amount of nitrogen functionalities revealed by energy dispersive X‐ray spectroscopy (XPS) and proper Fe−N_x_ site formation. HNO_3_ oxidation for 2 h leads to Fe−N−C with slightly lower oxygen reduction reaction (ORR) activity and selectivity. In contrast, the Fe−N−C synthesized from CA with H_3_PO_4_ treatment shows negligible ORR activity. The feasibility of one‐step activation and carbonization treatment with K_2_CO_3_ and, for the first time, with K_2_CO_3_ and melamine is proven as the obtained Fe−N−Cs exhibit promising ORR activity. The results are compared with the commercial Fe−N−C PMF‐014401. This study contributes to the advancement of cost‐efficient HT‐PEMFCs by optimizing Fe−N−C catalyst properties.

## Graphical abstract

Carbon aerogels are investigated as novel carbon support for FeN−Cs, which should serve as low‐cost cathode catalysts for high‐temperature PEMFCs. Examination in H_3_PO_4_ electrolyte reveals that various aerogel treatments affect oxygen reduction reaction activity and selectivity depending on surface functionalization. The fabricated carbon aerogel Fe−N−Cs are stable against peroxides.

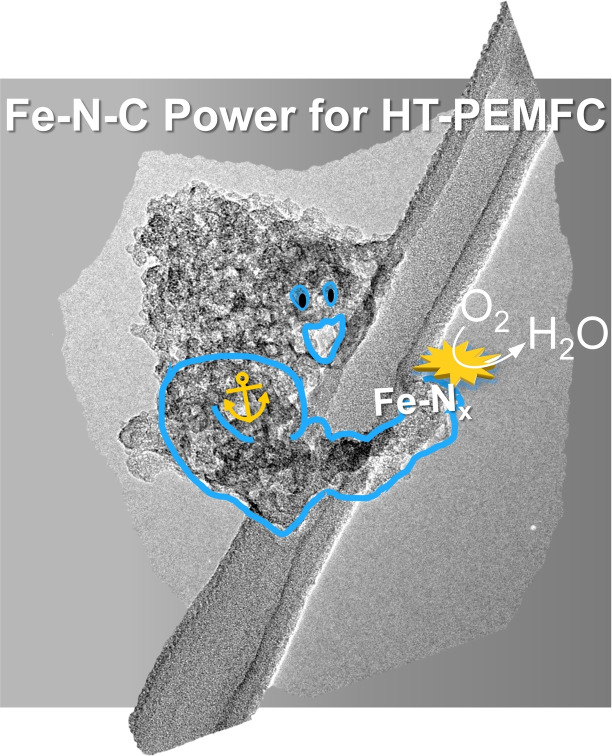

## 1 Introduction

Operating at approximately 160 °C, the HT‐PEMFC presents a promising technology for heavy‐duty, aircraft, and maritime applications.^[1]^ The possibility of air cooling, easier heat and water management, reduced periphery and weight savings give the HT‐PEMFC advantages over low‐temperature (LT)–PEMFCs for aviation.[^1^, ^2^] The superior CO tolerance of the HT‐PEMFC allows the utilization of (renewable) methanol and liquefied natural gas (LNG) in maritime applications.^[3]^

The catalyst of the HT‐PEMFC anode and cathode electrodes, typically consists of Pt‐ or Pt‐alloy nanoparticles supported on carbon such as carbon black (e. g. Vulcan®, Black Pearls^®^), graphitized carbon black or carbon nanotubes.^[5]^ However, phosphate ions from the phosphoric acid doped polybenzimidazole membrane partially poison the Pt surface,^[6]^ necessitating higher Pt loadings of up to 1 mg_Pt_ cm^−2^ per electrode, significantly increasing material costs. In contrast, LT‐PEMFCs require lower Pt loadings of approx. 0.4 mg_Pt_ cm^−2^.^[6]^

To reduce costs, alternative materials such as Pt‐free metal‐nitrogen‐carbon (M−N−C) catalysts are under investigation, with Fe−N−C being the most promising. This catalysts has a comparable ORR activity to Pt/C in acidic electrolyte[^1^, ^5^, ^7^] and remain active in diluted phosphoric acid (H_3_PO_4_)[^8^, ^9^] or in presence of added H_3_PO_4_ considering this electrolyte effect for HT‐PEMFCs.^[11]^ Contrary to Pt–based catalysts, Fe−N−Cs are not affected by phosphate poisoning, it is shown that phosphate adsorption on the Fe−N−C catalyst surface enhances the activity by supplying protons.[^1^, ^8^, ^13^, ^14^] Despite their sufficient ORR activity, the main challenges for Fe−N−C catalysts in PEMFC applications remain their low stability and mass activity.

Fe−N−C catalysts are typically fabricated by pyrolyzing nitrogen, carbon and iron containing precursors using template‐based, carbon support‐based or metal‐organic framework (MOF) synthesis approaches.

In carbon support‐based synthesis, selecting materials with suitable porous structures – including optimized surface area, pore size, and morphology – is critical,^[15]^ as these parameters can significant affect the catalyst activity^[16]^ and reactants mass transport.^[17]^ Among potential supports, carbon aerogels (CAs) are particularly promising due to their tunable porous structure, achievable through sol‐gel synthesis and subsequent drying and carbonization processes.[^16^, ^18^, ^19^] This flexibility in design, along with their scalability and serving as low‐cost carbon support makes CA an attractive option.[^16^, ^18^, ^19^]

Few demonstrations of CAs as supports for Fe−N−C catalysts in LT–PEMFCs emerged the past decade.[^19^, ^20^, ^21^] However, the requirements for HT‐PEMFCs, which involve a different operating environment remained unexplored yet.

Prior to the introduction of carbon supports into Fe−N−C synthesis, it is essential to incorporate surface functionalities (heteroatoms) into the carbon structure. Functionalities act as metal ion coordination sites,[^15^, ^16^, ^18^] stabilize the carbon network during electrochemical investigations^[24]^ and can enhance catalytic activity and stability by altering the electronic configuration.^[16]^ The amount, location and coordination type of the functional groups near the Fe−N_x_ sites influence the catalysts mass activity.[^15^, ^16^, ^18^] Moreover, the local carbon structure and pore connectivity to the electrolyte affect the catalyst performance.^[20]^ The tunable porous structure of CAs makes them highly adaptable to such requirements.[^16^, ^18^, ^19^] Consequently, detailed investigations into the pore structure and functionalization of CAs through different activation methods are needed for advancing Fe−N−C catalyst development for HT‐PEMFCs. The traditional method of activating carbons is chemical etching, where certain acids are used to create functionalities and increase the carbon surface area. In literature chemical KOH activation is reported to form K_2_CO_3_, decomposing to metallic potassium and CO_2_ above 700 °C which increases the porosity of the carbon network. Simultaneously, metallic potassium is intercalated into the carbon and further expands the framework.[^15^, ^16^, ^25^] Melamine is reported to introduce nitrogen functionalities into the carbon network.[^16^, ^20^]

In our study differently activated CAs are employed for Fe−N−C synthesis. Material structure and composition are analyzed and correlated with the electrochemical ORR activity, selectivity and stability in phosphoric acid electrolyte and compared with a commercial Fe−N−C (PMF‐014401, Pajarito Powder).

## 2 Results and Discussion

### 2.1 Physical Properties

**Figure 1:**
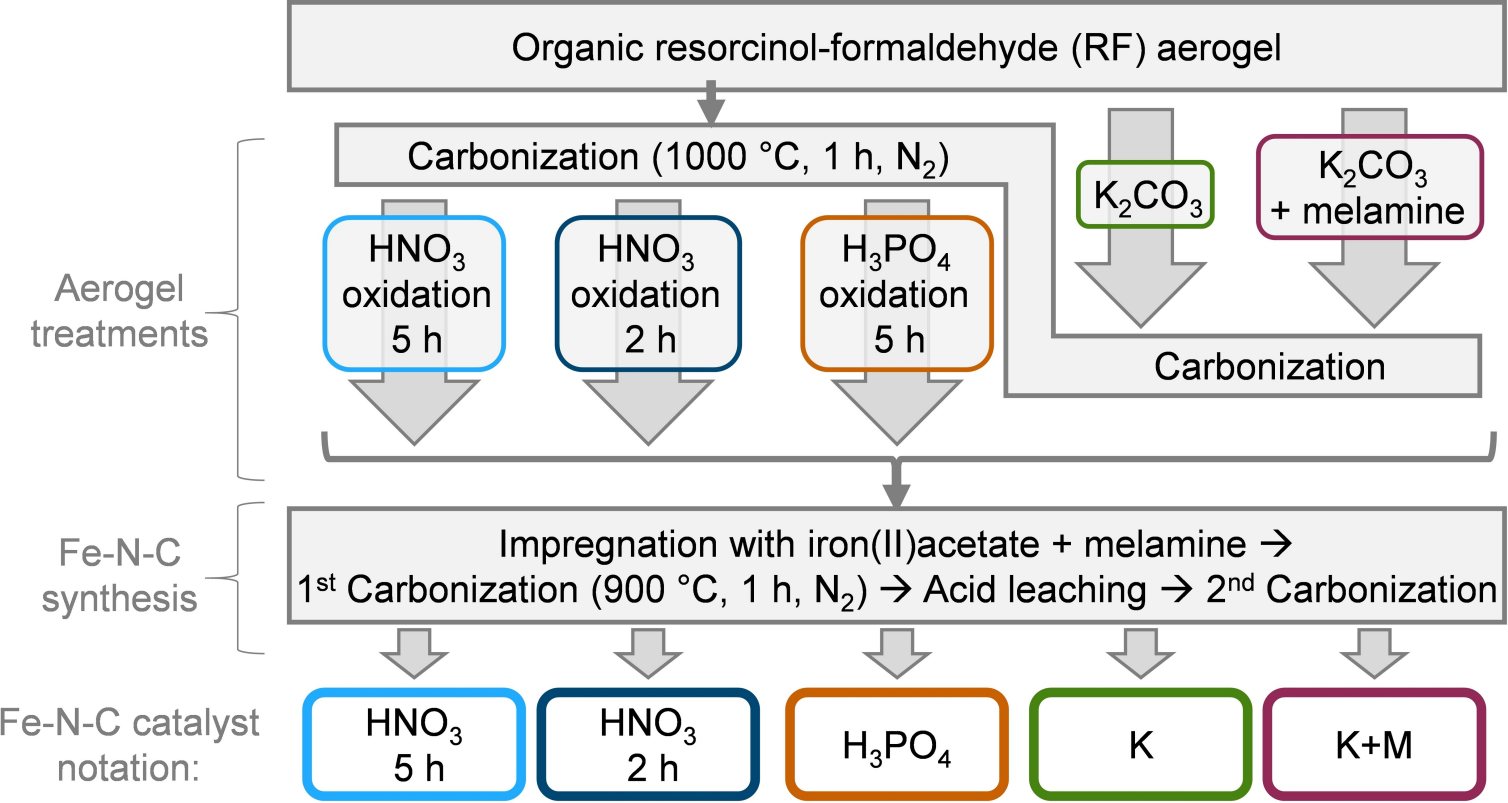
Schematic representation of the activation steps, Fe−N−C synthesis and catalyst notation.

A resorcinol‐formaldehyde (RF) aerogel is used as novel carbon support for Fe−N−C synthesis. The aerogel is prepared via sol‐gel process and activated using five different treatments as depicted in Figure 1. Functionalization of the RF aerogel is carried out by first carbonization to CA and then treatment with HNO_3_ for 2 h and 5 h and with H_3_PO_4_ for 5 h at 90 °C. By mixing K_2_CO_3_ (K) and K_2_CO_3_/melamine (K+M) with the organic RF aerogel prior to carbonization, activation and carbonization within one step is allowed.

**Figure 2:**
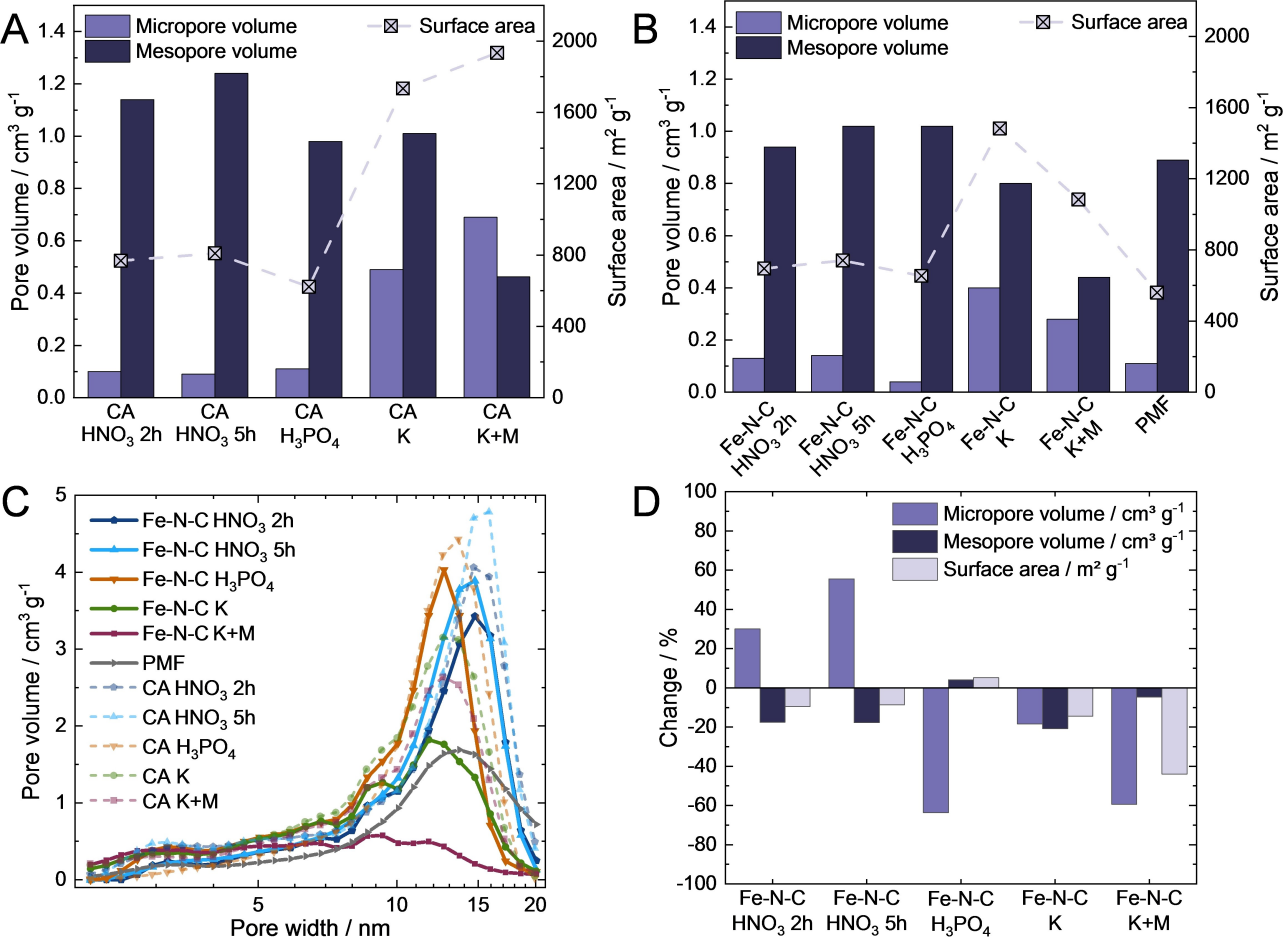
Pore volume and surface area of CAs (A), self‐synthesized Fe−N−Cs and PMF (B) and corresponding pore size distribution (C). Changes after synthesis from activated CA to corresponding Fe−N−C (D).

The pore types and volume of the CAs and the related Fe−N−Cs are analyzed by nitrogen sorption. All CAs in Figure 2‐A demonstrate surface area above 600 m^2^ g^−1^ and the presence of meso‐ and micropores, which are beneficial for Fe−N_x_ site incorporation during synthesis.^[15]^ The CAs have a significant higher surface area than typical carbon support like Vulcan with 100–300 m^2^ g^−1^.^[5]^ CA HNO_3_ 2 h demonstrates a slightly lower surface area compared to CA HNO_3_ 5 h as the extended oxidation time leads to a higher mesopore volume. CA K and CA K+M offer a more than twice as high surface area and five times higher micropore volume compared to the other CAs (Figure 2‐A) due to CO_2_ formation during carbonization from K_2_CO_3_.^[25]^

Figure 2‐B shows that PMF has a surface area similar to Fe−N−C HNO_3_ 2 h and 5 h and Fe−N−C H_3_PO_4_. The surface area (560 m^2^ g^−1^) and micro‐ and mesopore volumes (0.11 and 0.89 cm^3^ g^−1^) of the commercial PMF‐014401 are highly comparable to a comparable PMF‐011904 (590 m^2^ g^−1^; 0.103 cm^3^ g^−1^; 0.92 cm^3^ g^−1^) of Primbs et al.^[26]^, which shows reliable and comparable data in our study. Fe−N−C K has the highest surface area among the catalysts, followed by Fe−N−C K+M due to their large micropore volume.

The nitrogen sorption curves of CA and corresponding Fe−N−C are plotted in Figure 2‐C. For Fe−N−C HNO_3_ 2 h, Fe−N−C HNO_3_ 5 h and Fe−N−C H_3_PO_4_ pore volumes in the range of 10–20 nm decrease and shift to lower maximum pore width after synthesis, while the pore volume below approximately 8–10 nm increases. The pore volume of Fe−N−C K and Fe−N−C K+M in the range of 10–20 nm decrease more drastically. It can be assumed that for both Fe−N−Cs HNO_3_ and Fe−N−C H_3_PO_4_ the precursors are predominantly incorporated inside the mesopores, whereas for Fe−N−C K and Fe−N−C K+M they are apparently distributed inside both meso‐ and micropores.

Figure 2‐D visualizes the pore volume and surface area changes due to the synthesis. For Fe−N−C HNO₃ 2 h and 5 h, the surface area decreases by 10 %, with the micropore volume increasing by 30 % and 56 %, respectively, and the mesopore volume decreasing by approximately 18 % for both. These changes may result from the formation of additional microporous carbon within the pores,^[8]^ the loss of surface functionalities, or the incorporation of precursors into mesopores.

In contrast, Fe−N−C H₃PO₄ exhibits a negligible 5 % increase in surface area but a drastic 64 % reduction in micropore volume. This significant loss is likely due to precursor incorporation or the instability of micropores during thermal treatment.

The surface area of Fe−N−C K decreases by 14 %, with both micropore and mesopore volumes reduced by approximately 20 %. The most pronounced effects are seen in Fe−N−C K+M, which experiences a 44 % drop in surface area, driven by a 59 % decrease in micropore volume and a 5 % reduction in mesopore volume (Figure 2‐D). These changes point to precursor migration into the pores or partial pore collapse during the thermal treatment.

**Figure 3:**
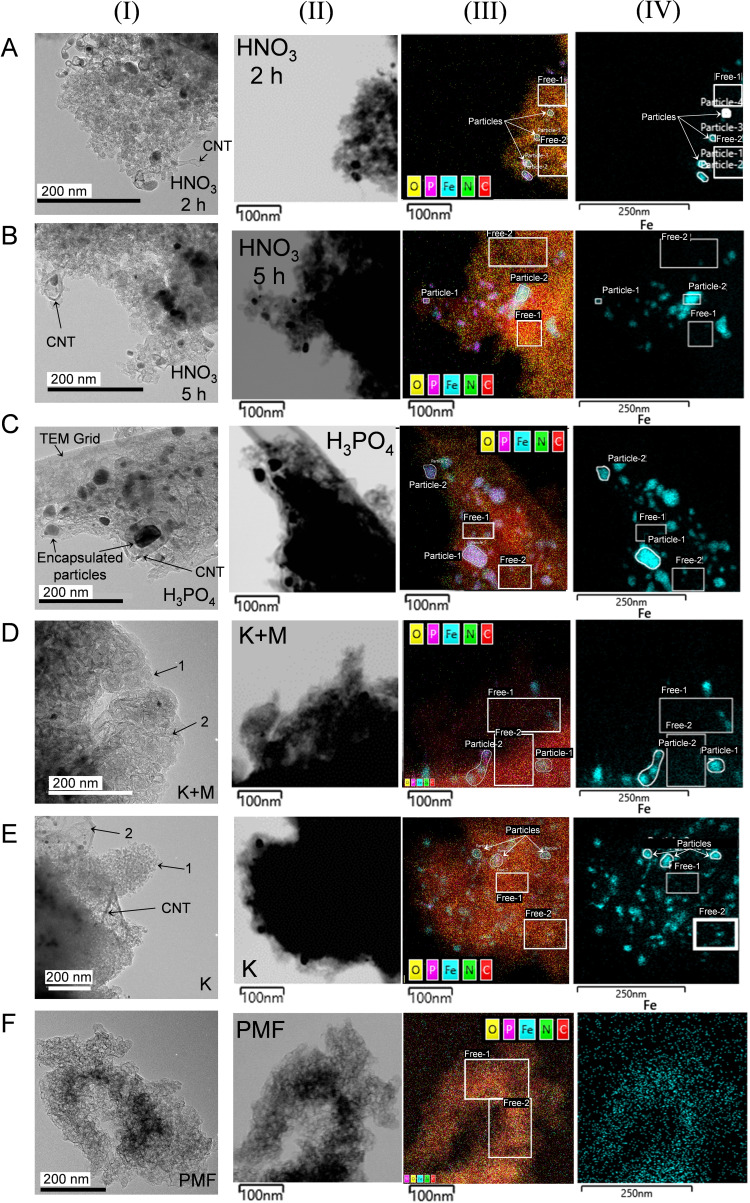
HR‐TEM images (I) and STEM images (II) of the Fe−N−Cs (A−E) and PMF (F). Corresponding elemental distribution map (III) and Fe map (IV), where the white framed boxes designate particle‐free regions (denoted as “Free”).

Nitrogen sorption analysis is complemented by high resolution (scanning) transmission electron microscopy (HR‐(S)TEM) (Figure 3 (I)) coupled with energy dispersive X‐ray spectroscopy (EDS) (Figure 3 (II)‐(IV)) to elucidate the catalyst morphology and elemental distribution. The HR–TEM images show that Fe−N−C HNO_3_ 2 h (Figure 3 (I)‐A) and Fe−N−C HNO_3_ 5 h (Figure 3 (I)‐B) consist of agglomerates with dimensions of several hundred nanometers and carbon nanotubes (CNTs) marked with arrows. The Fe maps in Figure 3 (IV) disclose iron‐rich particles inside the CNTs, which is reasonable as metallic iron particles are known to catalyze CNT formation.[^15^, ^27^] The particles show significantly more pronounced iron peaks than the particle‐free regions in the EDS spectra (Figure S1‐A and ‐B). The combined EDS mapping of C, N, O, P, Fe (Figure 3 (III)) illustrate the desired homogeneous distribution of Fe, N and C along the catalysts, indicating the homogenous incorporation of the precursors. EDS spectra (Figure S1‐A and ‐B in the supporting information) also reveal distinct iron peaks within the particle‐free regions.

Fe−N−C H_3_PO_4_ consists of agglomerates with dimensions of several hundred nanometers and few CNTs, similar to Fe−N−C HNO_3_ 2 h and 5 h. Additionally encapsulated particles are observed, which are marked in Figure 3 (I)‐C. For Fe−N−C H_3_PO_4_, insignificant amounts of phosphorus are detected in the particle‐free regions, so that the P doping of the carbon structure is negligible. However, the encapsulated particles are enriched in phosphorous and iron (Figure 3 (III)‐C and Figure S1‐C). Therefore, it is reasonable that fewer CNTs are observed for Fe−N−C H_3_PO_4_ as the particles consists of an iron‐phosphorous compound, compared to the Fe−N−C HNO_3_ catalysts where metallic iron particles have catalyzed the CNT formation.[^15^, ^27^]

In Figure 3 (I)–D and ‐E, the catalysts K and K+M appear to have, in addition to the carbon‐based agglomerates (marked with 1), a sheet‐like structure (marked with 2), which seems to be non‐porous. These second phases are also visible in the scanning electron microscopy images (Figure S2, supporting information). The structure has a similar appearance as exfoliated graphite and a graphene‐like morphology as seen in the TEM images of Schonvogel et al.^[28]^ It is expected that this structure results from intercalated potassium in the carbon[^15^, ^16^, ^25^] and occurrence is traced back to K_2_CO_3_ treatment. Homogenous distribution of C, N and Fe within the particle‐free carbon structure indicate the successful incorporation of the precursors.

The iron‐enriched particles in the self‐synthesized catalyst are protected by a graphitic shell (CNT), making their removal via acidic leaching difficult, as seen in other studies.^[15]^ Choi et al. observed leaching of iron particle from their encapsulated shell below 0.7 V in ring‐disk electrode experiments in acidic electrolyte, which can catalyze H₂O₂ and negatively affecting stability, but found no significant decrease in Fe−N−C stability.^[29]^ The commercial PMF catalyst in Figure 3 (I)‐F does not show any particles, due to its template‐based synthesis.[^6^, ^30^] The EDS spectra of PMF shows a distinct silicon peak (Figure S1‐F, in the supporting information), which is likely derived from the template.

In summary, for all self‐synthesized catalyst incorporation of the precursors into the carbon structure seem successful and homogenous.

EDS is complemented by the full quantification of the iron bulk content of the catalysts by inductively coupled plasma mass spectrometry (ICP‐MS) shown in Table 1. All catalysts have iron contents in reasonable range, underlining the EDS results. PMF has a lower iron content compared to the other catalysts. Literature has shown that increasing the iron content for Fe−N−Cs leads to an increase in performance, which then decreases after exceeding the optimal iron content of approx. 1.3–1.4 wt.% for LT–PEMFC applications.[^20^, ^31^] However, ICP–MS iron content cannot be directly correlated to ORR activity, since it does not differentiate between active Fe−N_x_ sites and inactive iron particles.

**Table 1:** Iron content of the catalysts determined by ICP‐MS.

Fe−N−C	HNO_3_ 5 h	HNO_3_ 2 h	H_3_PO_4_	K	K+M	PMF
Iron content/ wt_Fe_%	1.3	1.3	0.9	1.7	1.5	0.5

**Figure 4:**
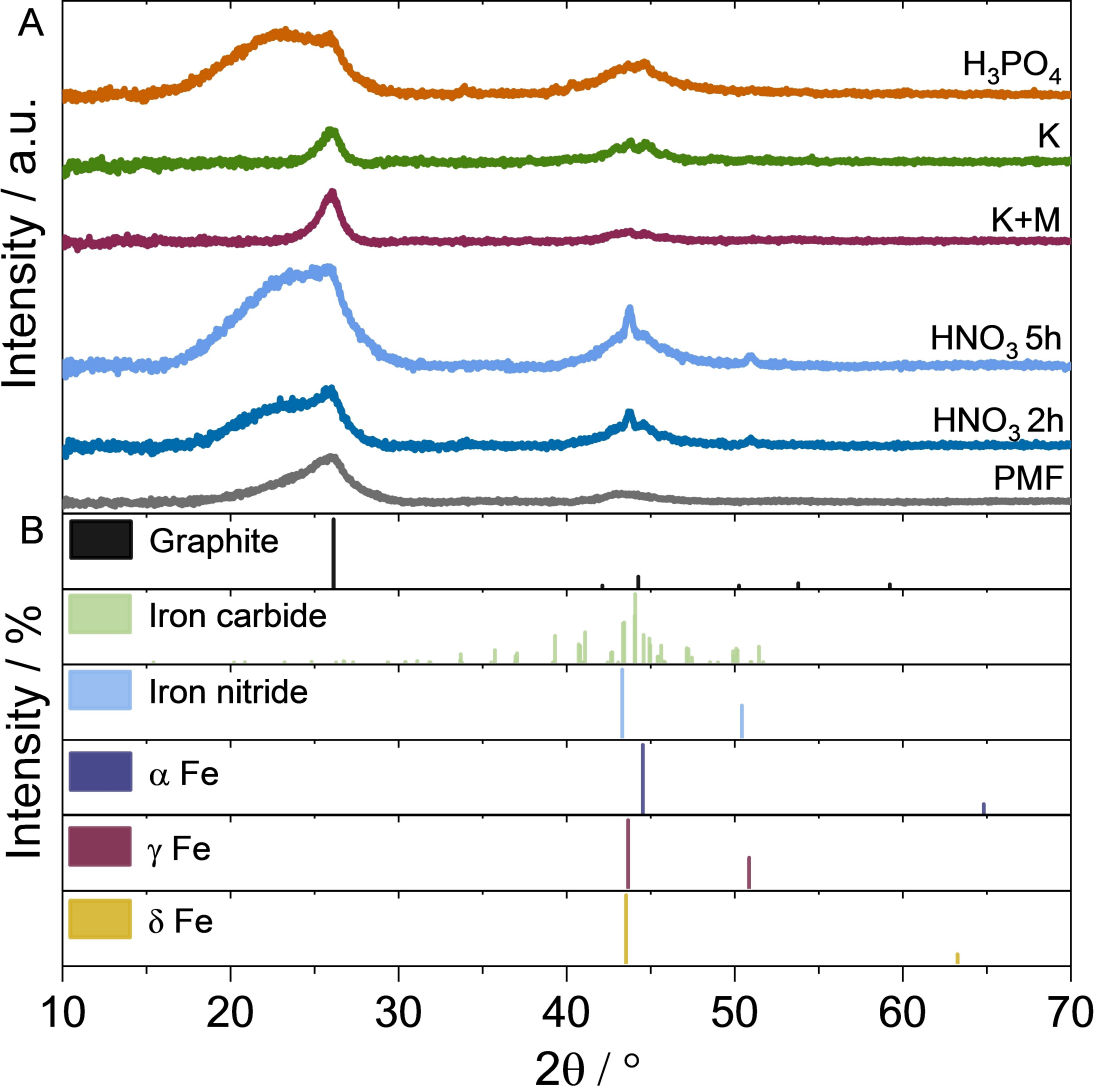
Diffractograms from XRD analysis of the self‐synthesized Fe−N−Cs and PMF (A). Patterns of reference signals are shown according to their peak intensity, where the highest peaks represent 100 % (B).

To further analyze the iron species and the carbon structure X‐Ray diffraction analysis (XRD) measurement was performed. Typical graphitic carbon peak (26°, 002; ICSD 98‐005‐3781 in Figure 4‐B) is found for all catalysts in Figure 4‐A.[^15^, ^32^] Fe−N−C H_3_PO_4_, Fe−N−C HNO_3_ 2 h and Fe−N−C HNO_3_ 5 h have a broad peak at 15–27°, which is not found for to Fe−N−C K and Fe−N−C K+M. This is attributed to a less structured graphitic carbon that can be impacted by the presence of iron‐containing particles (as seen in Figure 3 (I)‐A–C) and Fe, O or N functionalities.[^18^, ^34^] CNTs are also known to increase the spacing between the graphitic carbon sheets^[28]^ and contribute to the broader peak. In comparison, a sharper peak for Fe−N−C K and Fe−N−C K+M around 26° indicates high degree of graphitization of the catalysts.[^15^, ^35^] In literature, a higher degree of crystallinity of the carbon support of Pt‐based catalyst has been found among other influences to positively influence the stability against carbon corrosion.[^28^, ^36^]

Furthermore, the XRD patterns of all catalysts in Figure 4‐A show multiple peaks at around 45°, which can be traced back to graphitic carbon (44°; 100; ICSD 98‐005‐3781; Figure 4‐B). Additionally, metallic iron species are identified in the XRD patterns of the Fe‐N−Cs (α Fe, ICSD 98‐015‐9352; γ Fe, ICSD 98‐018‐5742 and δ‐Fe, ICSD 98‐005‐3452; Figure 4‐B), which is in line with the EDS results. In Figure 4‐A multiple peaks are present at 41–47° for the catalysts, but no distinct iron carbide (ICSD 98–024–533; Figure 4‐B) signals between 33–41° or at other possible reflexes are visible. Therefore, the presence of iron carbide is unlikely, but cannot be excluded by only XRD analysis. An equivalent assumption is made for iron nitride (ICSD 98‐003‐1901; Figure 4‐B), due to the overlap and low peak intensity, it is difficult to clearly assign the peaks.

XPS was performed to further analyze the Fe and N composition by investigating the near surface elemental composition and chemical state of the catalysts. The C1s and O1s spectra (Figure S3 in the supporting information) show no unexpected variances.

**Figure 5:**
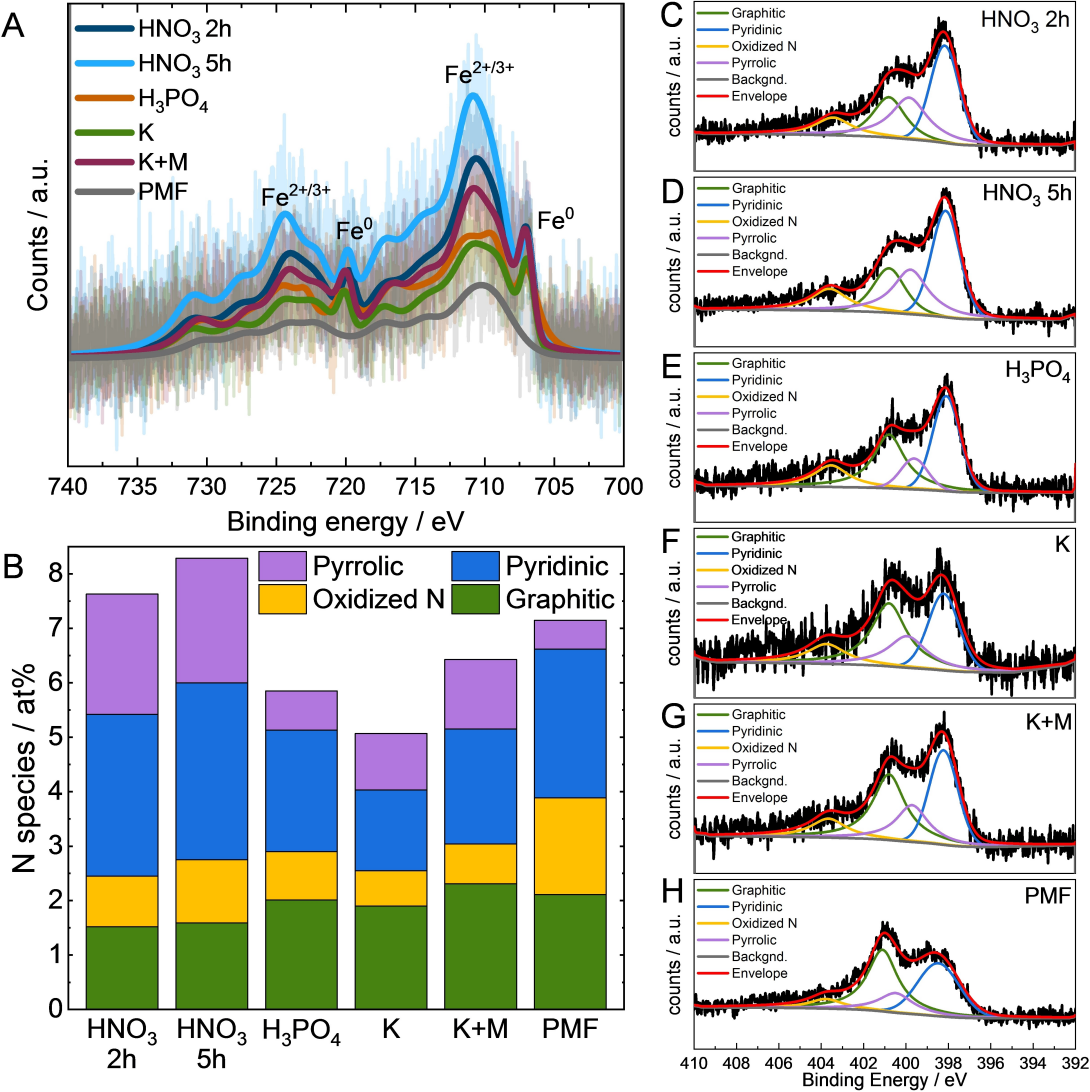
Background subtracted XP Fe2p spectra and envelope for the Fe−N−Cs (A). N1s spectra deconvolution for total N content and of N species content (B). XP N1s spectra of the Fe−N−Cs (D−H) and PMF (I).

Low iron amounts of 0.9–1.7 wt.% (ICP‐MS iron contents in Table 1) result in low Fe2p peak intensities of the XPS in Figure 5‐A, so that fitting of the data would be inadequate.[^15^, ^37^] However, Fe^2+/3+^ species are identified in the spectra. This species might be partly present as Fe−N_x_ sites.[^15^, ^38^, ^39^] Also, Fe^0^ peaks are noticed which is in agreement with the XRD and EDS results.

Figure 5‐B deconvolves the total N contents and N species of the Fe−N−C N1s spectra, which are depicted in Figure 5‐C to ‐H. The peak fitting was conducted according to Sadezky et al.^[40]^ First, the total N content is discussed along all catalysts. The Fe−N−C HNO_3_ 2 h and 5 h have the highest total N contents of 7.6–8.3 at % (Figure 5–B), as these catalysts already had N functionalities previous to the Fe−N−C synthesis. The lower N content of 5.8 at % for H_3_PO_4_ is reasonable because N functionalities are not expected to be incorporated during CA treatment, only P or O functionalities are expected, which then should serve as N anchoring points. The lower total N content for K (5.1 at %) compared to K+M (6.4 at %) is reasonable because utilized melamine has a high amount of N functionalities incorporated during the aerogel treatment. Overall the self‐synthesized catalysts have comparable N contents, while Fe−N−C HNO_3_ 2 h and 5 h and Fe−N−C K+M have slightly higher N contents as it is already introduced during the aerogel treatment.

The graphitic, oxidized, pyridinic and pyrrolic N contents (Figure 5‐B) are extracted from XP N1s spectra of the Fe−N−Cs (Figure 5‐C−H). In Figure 5‐B, slightly lower graphitic N contents are present for Fe−N−C HNO_3_ 5 h and HNO_3_ 2 h, compared to the other catalyst. Non‐Fe‐coordinated graphitic N can be responsible for the unfavorable two‐electron pathway.[^37^, ^41^] Therefore, lower graphitic N contents are preferred. According to Figure 5‐B, the order of cumulative pyridinic and pyrrolic N content is Fe−N−C HNO_3_ 5 h>HNO_3_ 2 h>K+M>PMF>H_3_PO_4_>K, while the contents are in a similar range of 5.5>5.2>3.4>3.3>3.0>2.5 at %. Higher contents of pyridinic and pyrrolic N may demonstrate higher amounts of active sites amounts which would benefit ORR activity.[^15^, ^38^, ^39^] However, if and how much pyridinic and pyrrolic N is connected to Fe cannot be clearly distinguished due to too low Fe2p intensities. While pyrrolic N (non‐Fe‐coordinated) is known to catalyze H_2_O_2_ which can negatively impact the catalysts stability[^37^, ^41^, ^42^, ^43^] the pyridinic N can reduce the unwanted H_2_O_2_ to H_2_O.[^43^, ^44^]

The physical analysis found a decrease of pore volume after synthesis for most of the catalysts, which attributed to precursor incorporation. HR‐TEM/EDS reveals evenly distributed iron for all catalysts and some iron‐rich particles, and CNTs, particularly in Fe−N−C HNO_3_ 2 h and 5 h and Fe−N−C H_3_PO_4_. XRD analysis confirms the presence of iron species for all catalysts and less organized carbon structure due to functionalities and particles. XPS indicates the highest pyridinic and pyrrolic nitrogen content in Fe−N−C HNO₃ (2 h and 5 h), followed by Fe−N−C K+M, Fe−N−C K, and Fe−N−C H₃PO₄. Pyridinic and pyrrolic N are found in all catalyst, which can be coordinated to Fe or are present as Fe‐free species.

### 2.2 Electrochemical Activity, Selectivity and Stability

The ORR activity, selectivity and stability of the Fe−N−Cs are investigated in diluted phosphoric acid within a rotating ring‐disk electrode (RRDE) setup to determine their suitability as a cathode catalyst for HT‐PEMFCs. For stability evaluation an accelerated stress test (AST) consisting of square‐wave potential cycling with 10,000 sweeps between 0.6 and 1.0 V (3 s each) in O_2_ saturated electrolyte is employed. This harsh stress test operates in actual potential ranges of PEMFC operation and targets degradation induced by reactive oxygen species (ROS)^[45]^ and subsequent demetallation of Fe−N_x_ sites and also electrochemical carbon corrosion.[^29^, ^47^]

**Figure 6:**
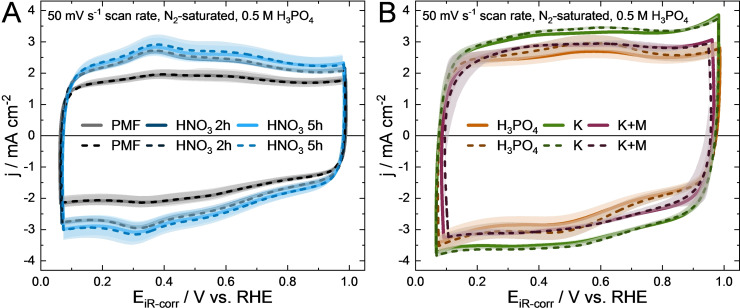
CV curves of the PMF, Fe−N−C HNO_3_ 2 h and 5 h (A) and of the Fe−N−C H_3_PO_4_, K and K+M catalysts (B) before (solid lines) and after (dashed lines) 10,000 potential square wave cycling AST between 0.6 and 1.0 V (O_2_‐satured electrolyte during AST). Mean current densities are plotted and the shading around the curves illustrates the standard deviation.

First, the cyclic voltammograms (CVs) of the Fe−N−Cs are examined. Redox peaks at 0.2–0.4 V are evident for Fe−N−C HNO_3_ 2 h, Fe−N−C HNO_3_ 5 h and PMF (Figure 6‐A). Similar peaks have been observed in our previous studies and can be attributed to iron redox peaks.[^6^, ^24^, ^48^] This assumption is based on the findings of Wang et al., where they observed that the iron redox couple peak of Fe−N−C catalysts shifts from around 0.8 V in HClO_4_ to 0.62 V in H_2_SO_4_ electrolyte due to the weaker adsorption strength of the corresponding anion with iron.^[14]^ Since phosphate anions even have a lower adsorption strength than sulfate anions^[14]^, this likely explains the further peak shift to 0.2–0.4 V in our study.

For Fe−N−C H_3_PO_4_, Fe−N−C K and Fe−N−C K+M (Figure 6‐B), no iron redox peaks around 0.2–0.4 V are visible. They are either hidden by the capacitive currents or the accessibility of Fe−N_x_ is low as they might mainly be incorporated in micropores for Fe−N−C K and Fe−N−C K+M (Figure 2). However, the occurrence of Fe redox peaks in CVs is not a measure for proper ORR activity.[^6^, ^49^] Fe−N−C H_3_PO_4_ shows additional redox peaks between 0.5–0.6 V in the CVs (Figure 6‐B), which can be attributed to hydroquinone/quinone species and/or Fe^2+^/Fe^3+^ redox transitions.^[6]^

Overall all self‐synthesized Fe−N−Cs seem to have similar accessible electrochemical double layer as the differences in the CVs are minimal. Slightly higher capacitive currents are observed for Fe−N−C K (Figure 6‐B), which might be attributed to the catalyst high surface area (Figure 2‐B). Higher surface area of the carbon structure is wetted by electrolyte, leading to higher capacitive current densities.^[6]^ A similar effect is observed for PMF.

During the AST, carbon corrosion can induce an increase in capacitive currents due to the carbon surface oxidation.[^24^, ^41^] No distinct changes are observed for the CVs before and after the AST in Figure 6. This is similar to the double layer capacity (DLC), which is calculated at 0.2 and 0.8 V (Figure S4 in the supporting information). The slight increase of redox peaks between 0.5–0.6 V for Fe−N−C H_3_PO_4_ after the AST in Figure 6–B might be attributed to formation of hydroquinone/quinone species due to partial surface oxidation. As the capacitive currents in the CVs remain unchanged after AST for all catalysts the stability is high under the harsh AST conditions in relevant potential range under oxygen.

**Figure 7:**
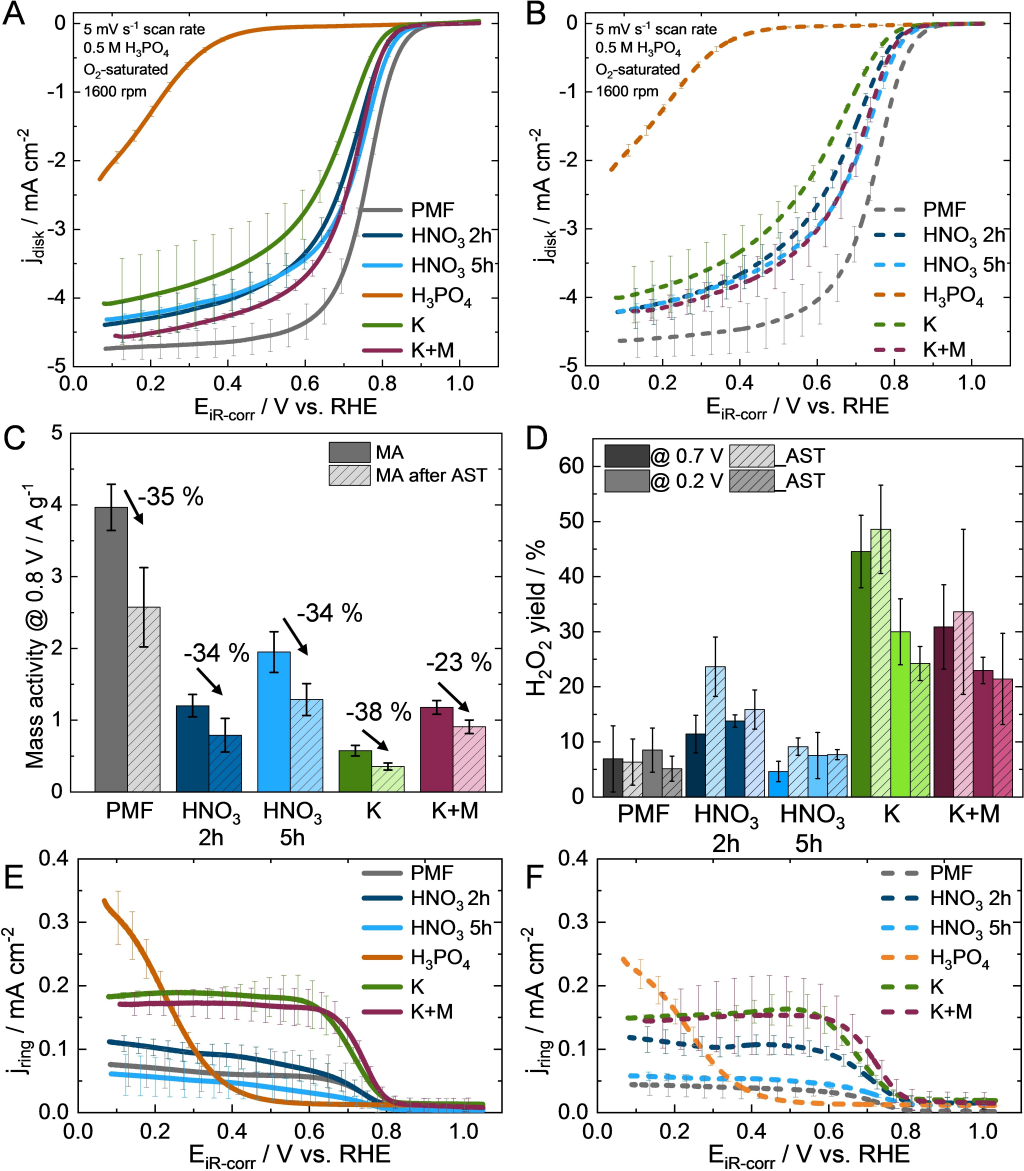
ORR polarization curves with standard deviation from three measurements before (A) and after (B) 10,000 potential square wave cycling AST between 0.6 and 1.0 V (O_2_‐satured electrolyte during AST). Mass activities for the ORR at 0.8 V (C). Mean values of the H_2_O_2_ yields at 0.2 V and 0.7 V (D) from ring current densities with standard deviation from three measurements before (E) and after (F) AST.

Next, the ORR activity is evaluated via the polarization curves (Figure 7‐A). Fe−N−C HNO_3_ 5 h and 2 h and Fe−N−C K+M display highly comparable polarization curves, while Fe−N−C HNO_3_ 2 h and 5 h exhibit higher currents within the kinetically controlled region (>0.9 V) and diffusion‐kinetically mixed region (0.7–0.9 V) than Fe−N−C K+M, which inverts within the diffusion limited region (<0.7 V). Slightly lower activity is demonstrated by Fe−N−C K. In contrast, Fe−N−C H_3_PO_4_ shows insignificant activity towards ORR. The highest ORR activity is detected for PMF as the lowest overvoltage is achieved. Figure 7‐B reveals only a small decrease in ORR activity after the AST for all Fe−N−Cs compared their initial activity (Figure 7‐A), which again demonstrates a high stability of the catalysts.

The highest mass activity (MA) at 0.8 V before the AST in Figure 7‐C for the Fe−N−Cs are observed in the following order: PMF>HNO_3_ 5 h>HNO_3_ 2 h≈K+M>K. This order remains also after AST as the catalysts show comparable MA losses in the range from 23 to 38 %. Fe−N−C H_3_PO_4_ is excluded from the MA calculations, as no reliable diffusion‐limited current densities can be extracted from the curve, and this catalyst is not suitable for catalyzing ORR.

Figure 7‐D depict the H_2_O_2_ yields, which are calculated according to literature^[26]^ at potentials of 0.2 and 0.7 V using the ring and disc current density curves (Figure 7‐A, ‐B, ‐E and ‐F). Fe−N−C H_3_PO_4_ is excluded as the calculation would not lead to a reasonable comparison. PMF and Fe−N−C HNO_3_ 5 h have the lowest H_2_O_2_ yield (<10 %) and thus the highest selectivity towards the favorable four‐electron ORR pathway. They are closely followed by Fe−N−C HNO_3_ 2 h. A significant proportion of Fe−N−C K and K+M catalyze the unfavorable two‐electron pathway, leading to high H_2_O_2_ yields of 20–50 % and demonstrate an inferior selectivity compared to the Fe−N−Cs treated with HNO_3_ (Figure 7‐D).

PMF, Fe−N−C K and K+M reveal no significant change in H_2_O_2_ yields before and after AST (Figure 7‐D). The increased H_2_O_2_ yield of Fe−N−C HNO_3_ 5 h and Fe−N−C HNO_3_ 2 h at 0.7 V after AST, indicate an increased ratio of catalyzing oxygen via the two‐electron pathway, as H_2_O_2_ is an intermediate. Both catalysts H_2_O_2_ yields are unchanged at 0.2 V after the AST. It is noteworthy, that the overall selectivity of the Fe−N−C HNO_3_ 5 h is highly comparable to that of PMF. For all Fe−N−Cs no drastic change in the H_2_O_2_ yields after the AST is observed.

In Figure 7‐E, ring current density curves of Fe−N−C HNO_3_ 2 h and 5 h as well as for the PMF start with no/negligible current density flow at 1.0 V. The ring current density increases slightly from 0.8 to 0.6 V and afterwards reaches a stable plateau in the diffusion limiting region, which is in line with the ORR in the polarization curves (Figure 7‐A). A similar curve shape is observed for Fe−N−C K and K+M with higher current densities in the range of 0.8–0.1 V. In comparison, Fe−N−C H_3_PO_4_ has a completely different ring current shape and shows an exponential current increase from 0.4–0.1 V in Figure 7‐E. The ring currents increase where the ORR curve at approx. 0.4 V starts (Figure 7‐A).

For PMF, an insignificantly decrease in ring current densities is observed after the AST (Figure 7‐F). The ring current density of Fe−N−C HNO_3_ 2 h increases slightly and exclusively in the range of 0.3–0.7 V. Fe−N−C HNO_3_ 5 h, Fe−N−C K and K+M reveal no distinct change in ring current density curves after AST. The ring current density (Figure 7‐F) of Fe−N−C H_3_PO_4_ catalyst still increases steeply after 0.4 V, but to a lesser extent.

Analysis of the Tafel plots (in Figure S5 in the supporting information) show highly comparable slopes for the self‐synthesized catalyst (range of 73–75 mV dec^−1^ (before AST), 80–85 mV dec^−1^ (after AST)), which are within the typical Tafel slope ranges of 60–80 mV dec^−1^ for Fe−N−Cs in acidic media.[^18^, ^20^, ^41^, ^50^] The modest increase in the Tafel slope can indicate a change in the rate‐determining step or oxidation of the carbon surface during the AST.^[41]^ PMFs slope of 66±1 mV dec^−1^ indicating a more effective pathway for ORR which is in line with the highest MA changes and changes to 69 mV dec^−1^ after AST.^[18]^

### 2.3 Correlation of Physicochemical Properties with ORR Activity, Selectivity and Stability

In this section the ORR activity, selectivity and stability are linked to the physicochemical properties of the CA‐based Fe−N−C catalysts and related to their activation methods. The similarities and differences between the catalysts are compared among each other.

#### 2.3.1 Effect of HNO_3_ Treatment

Fe−N−C HNO_3_ 5 h has the highest MA and selectivity among the self‐synthesized catalysts. The MA of the Fe−N−C HNO_3_ 2 h is 38 % lower than Fe−N−C HNO_3_ 5 h. On the one hand, this could be attributed to the slightly lower pyridinic and pyrrolic N content of Fe−N−C HNO3 2 h by 6 % (0.3 at %), and more importantly less amounts appearing to be coordinated to Fe. This is evidenced by the 45 % higher H_2_O_2_ formation rate of Fe−N−C HNO_3_ 2 h compared to Fe−N−C HNO_3_ 5 h. However, given that no extensive high H_2_O_2_ yields (below 20 % before AST) are observed for both Fe−N−C HNO_3_ 2 h and 5 h, it can be postulated that the majority of pyridinic and pyrrolic N is coordinated to iron. Otherwise, the two‐electron pathway would result in significant higher ring current densities, which is not the case (Figure 7‐E). On the other hand, the twice as much increased micropore volume after treatment with HNO_3_ for 5 h compared to 2 h indicates the formation of a larger number of defects which can host active sites during Fe−N−C synthesis. In conclusion, the CA oxidation with HNO_3_ must take place for at least 5 h to generate beneficial surface composition and larger surface area (Figure 2) and allow a significant amount of Fe−N_x_ site formation during synthesis.

#### 2.3.2 Effect of K_2_CO_3_ and Melamine Treatment

Fe−N−C K has a 70 % lower MA than Fe−N−C HNO_3_ 5 h and exhibits the lowest ORR activity and selectivity among the catalysts (excluding Fe−N−C H_3_PO_4_), which can be traced back to three aspects. First, a more than 50 % lower pyridinic and pyrrolic N content of 2.5 at % (Figure 5‐B), compared to the Fe−N−Cs HNO_3_ which can be a sign for lower amount of active Fe−N_x_ sites. The treatment with only K_2_CO_3_ seem to not establish enough anchor points for nitrogen and iron ions during synthesis. Second, less amount of N functionalities are coordinated to iron, which means fewer quantity of active Fe−N_x_ sites, as evidenced by the highest H_2_O_2_ formation rate and low MA. This is based on the findings, that N functionalities, which are not coordinated to iron, like graphitic N and pyrrolic N favor the two‐electron pathway and, as a consequence, increase the H_2_O_2_ yields.[^37^, ^41^, ^42^, ^43^] The third aspect is the approximately one‐third higher graphitic N content (Figure S6) in terms of total composition, than both Fe−N−C HNO_3._ Graphitic N can catalyze oxygen via two‐electron pathway and lead to higher H_2_O_2_ formation rate.^[51]^ Moreover, high H_2_O_2_ yields of Fe−N−C K might lead to demetallation of the active Fe−N_x_ sites and carbon corrosion due to the formation of ROS. This renders Fe−N−C K an unattractive option as ORR catalyst.

The treatment with K_2_CO_3_ and melamine leads to higher amount of anchor point during synthesis and higher MA of Fe−N−C K+M. The pyridinic and pyrrolic N content of Fe−N−C K+M (3.4 at %) is roughly a quarter higher than Fe−N−C K and correlates with a higher MA. At this point it is noteworthy to mention, that the amount of pyridinic and pyrrolic N content of the self‐synthesized catalysts (apart from Fe−N−C H_3_PO_4_) are on line with the MA trend (Figure S7 in the supporting information).

Beside beneficial surface functionalization, the high surface area of Fe−N−C K+M (Figure 2‐B) allows high accessibility from the electrolyte to the active Fe−N_x_ sites, which are located in the large micropores and small mesopores. In comparison to both Fe−N−C HNO_3_, where mainly mesopores host Fe−N_x_ sites, larger pores allow for a higher degree of electrolyte access to active sites, leading to high catalyst utilization. Lower selectivity of Fe−N−C K+M compared to both Fe−N−C HNO_3_ can be traced back to the same assumptions made for Fe−N−C K.

#### 2.3.3 Effect of H_3_PO_4_ Treatment

Although the pyridinic and pyrrolic N contents of Fe−N−C H_3_PO_4_, determined via XPS in Figure 5‐B, were comparable to those of the other catalysts, the iron content determined by ICP–MS (Table 1) was the lowest in comparison. Furthermore, EDS showed Fe‐/P‐containing particles. Thus, it can be concluded, that most of the pyridinic and pyrrolic N species are not coordinated to iron. Therefore, the two‐electron pathway is predominant and leads to a high peroxide formation rate.[^37^, ^41^] This is evidenced by the comparatively high ring current densities (Figure 7‐E). Additionally, below the potential of 0.4 V, encapsulated iron, as detected within HR‐TEM/EDS in Figure 3‐C, could leach out, catalyze H_2_O_2_ formation and further contribute to this exponential H_2_O_2_ increase.^[29]^ During the AST some of the particles or functionalities of Fe−N−C H_3_PO_4_ may be detached or leached due to ROS from the carbon structure leading to a lower H_2_O_2_ formation rate after AST.

#### 2.3.4 Stability

The MA at 0.8 V losses around 23–38 % after the AST are comparable for all investigated Fe−N−Cs (Figure 7‐C). Low stability for Fe−N−C K and Fe−N−C K+M would be expected due to their high H_2_O_2_ formation rate. However, this is not the case and could be attributed to first, the higher graphitic degree (Figure 4) of the carbon structure^[28]^ and second, the catalyzation of peroxides to water by pyridinic N, which is not coordinated to Fe.^[43]^ The stability of the CA based Fe−N−C catalyst systems against ROS during the harsh conditions of the 10,000 square cycles between 0.6 and 1.0 V under O_2_‐saturated electrolyte is remarkable high compared to other M−N‐Cs in literature. The ZIF‐8 based Fe−N−C of Kumar et al. shows a much higher loss of 66 % of MA after application of the same AST protocol in 0.1 M HClO_4_ with a catalyst loading of 800 μg cm^−2^.^[47]^ Further, a 51 % loss of kinetic current density at 0.8 V of Fe−N−C (from polypyrrole nanotubes as carbon support precursor) was found in the study of Gridin et al. for their state of the art Fe−N−C.^[52]^

In summary, similar ORR, selectivity and stability are achieved for all self‐synthesized, except for Fe−N−C H_3_PO_4,_ Fe−N−C catalysts and slight deviations of the catalysts are traced back to their aerogel modification.

## 3 Conclusions

The study utilizes the same type of aerogel in various treatment procedures. As one approach the aerogel is doped with K_2_CO_3_ (K) and a mixture of K_2_CO_3_ and melamine (K+M), followed by their carbonization. In comparison, the already carbonized aerogel is oxidized with HNO_3_ for 2 h and 5 h and H_3_PO_4_ for 5 h at 90 °C. These CAs are employed as novel carbon support precursors for Fe−N−C and disclose a less hazardous synthesis route compared to PMF, by replacing the hydrofluoric acid etching to remove the template, while remaining the advantage of tunable porous structure. For the first time, CA based Fe−N−Cs are investigated in diluted phosphoric acid as electrolyte to identify their potential as a cathode catalyst for HT‐PEMFCs.

- Higher pyrrolic and pyridinic N contents, incorporated during the treatments, are in line with higher ORR activity of the Fe−N−Cs.
- H_3_PO_4_ treatment is not beneficial, as the subsequent Fe−N−C demonstrates negligible activity towards ORR and by far the highest H_2_O_2_ yields.
- Fe−N−C K and Fe−N−C K+M reveal a significant lower selectivity, which is attributed to higher graphitic N content and lower pyrrolic and pyridinic N contents compared to Fe−N−C HNO_3_ (2 h and 5 h). Further, a second morphological phase, attributed to intercalated potassium during K_2_CO_3_ treatment is recognized.
- The oxidation time of the CA with HNO_3_ for 2 h appears incomplete to achieve similar nitrogen and iron ion anchor sites and surface functionalization compared to CA oxidation for 5 h, and results in lower ORR selectivity of the corresponding Fe−N−C HNO_3_ 2 h.
- Fe−N−C HNO_3_ 5 h shows the highest activity and selectivity among all Fe−N−Cs before and after the AST, only slightly lower than those observed for the commercial Fe−N−C (PMF).

The results highlight the significant influence of aerogel treatment on catalyst activity, stability and selectivity, emphasizing the crucial role of treatment procedures in optimizing Fe−N−C catalyst performance for this novel and cost‐efficient carbon support. Analysis of the most promising Fe−N−C HNO_3_ 5 h within GDE half‐cell or HT‐PEMFC single cell applications would be of interest to gain insight into more realistic performance. Upscaling of the synthesis and optimization of the K+M and HNO_3_ treatments should be a focus of future research to streamline the synthesis and increase the number of active sites. Moreover, other CA types, such as resorcinol‐melamine‐formaldehyde compositions, should be investigated as potential novel carbon supports. Our research paves the way for upscaling of the Fe−N−C synthesis and the one‐pot Fe−N−C synthesis, where Fe−N_x_ sites are integrated alongside with carbonization to economize the synthesis, for application in cathodes of HT‐PEMFCs.

## 4 Experimental Section

### 4.1 Synthesis

To synthesize the CAs, first the RF aerogel with a molar ratio of 1:0.74:0.038 : 250 (R : F : W : C) was prepared according to the following steps. Distilled water (W) was first weighed into a beaker glass. Resorcinol (R) (98 %, VWR Chemicals) was then added, and the solution was stirred, until the resorcinol had completely dissolved. Formaldehyde (F) (23.5 % solution, Carl Roth) was then added and stirred for a further 5 min. Next, Na_2_CO_3_ (C) (solid, Honeywell Chemicals, VWR) was added to the solution. The solution was stirred for 30 min at room temperature, poured into a tightly sealed container (glass or PP) and left to gel and aging in an oven at 60 °C for one week. Afterwards, the gels were removed from the oven, cooled to room temperature and carefully removed from the container and placed in a container filled with acetone. The acetone was replaced twice a day. After three days of washing, the washed gel was supercritical dried in an autoclave with supercritical CO_2_ (100 bar, 60 °C, mass flow rate 15 to 30 kg h^−1^).

For the CAs denoted as CA K and CA K+M, the resorcinol‐formaldehyde aerogel pieces were grinded in a 50 mL steel container (three 15 mm balls for 30 seconds at 30 Hz) with potassium carbonate (K_2_CO_3_, pure, Merck) and melamine (99 %, Thermo scientific) with a mass ratio of RF : K_2_CO_3_ 1 : 1 and RF : K_2_CO_3_ : Melamine 1 : 1 : 1. Subsequent carbonization for 1 h in nitrogen atmosphere (Linn High Term, 10 L/h nitrogen flow, 50 mbar) at 1,000 °C with a heating rate of 5 °C min^−1^ was conducted. To eliminate the potassium ions, the CAs were washed with distilled water until a neutral pH‐value of the washing solvent was reached. Afterwards, the CAs were ball milled in a 50 mL steel container (three 15 mm steel balls) for three times two minutes at 30 Hz.

For the CAs denoted as CA HNO_3_ (2 h and 5 h) and CA H_3_PO_4_ the RF aerogel were firstly carbonized under nitrogen atmosphere (Linn High Term, 10 L h^−1^ nitrogen flow, 50 mbar) at 1,000 °C with a heating rate of 5 °C min^−1^ for one hour, resulting in the CA. Afterwards the CA pieces were pulverized with a shaker mill (MM400, Retsch) in a 50 mL steel container (three 15 mm steel balls for three times two minutes at 30 Hz). To obtain oxygen and nitrogen‐doped CAs, 9 g of the pre‐ground CA were mixed with 75 mL of nitric acid (HNO_3_, 65 vol.%, AppliChem, VWR) in a round‐bottom flask and stirred at 90 °C for 2 h and for 5 h with a reflux condenser to produce CA HNO_3_ 2 h and CA HNO_3_ 5 h. Analogously, phosphoric acid (H_3_PO_4_, 85 vol.%, Honeywell) was used for 5 h to prepare the CA H_3_PO_4_. Afterwards, the CAs were ground under the same conditions as described before.

Our previously used support‐based Fe−N−C synthesis^[15]^ is adapted to this novel CA support material. For synthesis of Fe−N−C catalyst, 300 mg CAs were impregnated with a mixture of 33.9 mg iron(II)acetate (95 %, Sigma Aldrich) and 1,010.4 mg dry cyanamide (99 %, Sigma Aldrich) in 2 mL ethanol (99.8 %, Carl Roth). In sum, a ratio of 22.2 % CA, 74.9 % cyanamide and 2.9 % iron(II)acetate with ratios of 30.4 % C, 68.3 % N and 1.3 % Fe were used. The mixture was placed in a sonification bath (35 °C) until complete evaporation of ethanol and afterwards dried under reduced pressure over night at 30 °C in the vacuum drying oven (VDL 115, Binder). The powder was then milled with a mortar and pistil and placed into a ceramic boat. The boat was inserted into a tube furnace (RHTC 80‐230‐15, Nabertherm) and pyrolysis was carried out for 1 h at 900 °C with a heating rate of 5 °C min^−1^ and 100 L h^−1^ nitrogen flow rate. Then, the catalyst was inserted into a 500 mL round bottom flask equipped with a reflux condenser and a stir bar. 200 mL of 2 M H_2_SO_4_ (98 %, Carl Roth) were added and the dispersion was heated at 90 °C for 16 h. Afterwards, the acid was removed by filtration, using a membrane pump and a 0.05 μm membrane filter (Cytiva). The catalyst powder was washed with ultra‐pure water until neutral pH. Finally, the powder was dried under reduced pressure overnight at 30 °C, and a second pyrolysis analogous to the first pyrolysis was carried out.

### 4.2 Physical Analyses

Gas sorption experiments were carried out using a Micromeritics 3Flex instrument. The analysis adsorptive was nitrogen operating at 77 K. All samples were preconditioned at 200 °C for 12 h under vacuum (0.1–0.5 mbar) using a Micromeritics SmartVacPrep instrument. Pore width distribution was obtained using the density functional theory (DFT) with N_2_‐DFT model and specific surface areas were calculated using the Brunauer‐Emmett‐Teller (BET) method. For micropore volume t‐plot method and for mesopore volume Barrett‐Joyner‐Halenda (BJH) method was used with Carbon Black statistical thickness surface area (STSA) model.

Scanning electron microscopy images were recorded using a Zeiss Ultra 55 with 3.0 kV acceleration voltage.

Each catalyst was dispersed in ethanol in a ultrasonication bath and one drop (ca. 8 μL) deposited onto a Lacey carbon film with 400 meshes (Plano). For HR‐TEM imaging a JEM2100F (Joel GmbH) with 200 kV acceleration voltage was used, equipped with the 250 EDS X–Max80 SDD detector (Oxford Instruments). AZtec software (Oxford Instruments) was used for evaluation of EDS mapping.

Powder XRD measurements were performed using an Empyrean Series 2 diffractometer (PANanalytical) in Bragg‐Brentano geometry. Each catalyst was dispersed in 2‐propanol in an ultrasonic bath and dropped onto a zero‐background silica‐holder. After drying an even catalyst layer was formed. The measurements were conducted using Cu‐Κα radiation at a scan range between 5–70° with a voltage of 40 kV and a current of 40 mA. The resulting diffractograms were analyzed using the HighScore Plus (PANanalytical) software.

For XPS analysis an ESCALAB 250Xi (Thermo Fisher) was used with monochromatic Al‐Κα radiation and a beam diameter of 650 μm. Three survey scans were recorded, using a transit energy of 100 eV, a dwell time of 20 ms and a step size of 1 eV. Furthermore, high‐resolution spectra were recorded for the elements C (1 s) (3 scans), O (1 s) (5 scans), N (1 s) (10 scans) and Fe (2p) (10 scans) were recorded. A transit energy of 20 eV, a dwell time of 50 ms and a step size of 0.02 eV were used. The Avantage software (Thermo Fisher) was used with a smart background and Gauss‐Lorentz line shape for peak fitting.

To examine the iron content of the catalysts ICP–MS was conducted with the iCap or XSeries2 device (Thermo Fisher Scientific). 15 mg Fe−N−C catalyst was digested in 2 mL concentrated HNO_3_ (Rotipuran^®^Supra 69 wt %, Carl Roth) and boiled for 1 h at 100 °C. The solution was stored overnight. Afterwards, the sample was filtrated and the filtrate volume adjusted to 50 mL by addition of ultrapure water. 10 μL of a scandium internal standard (1,000 mg L^−1^, Carl Roth) were added to 10 mL of the sample solution. The calibration solutions consisted of a Fe ICP standard (Carl Roth) with concentrations of 0; 5; 10; 20; 500; 1,000; 2,000 and 3,000 μg L^−1^. A correlation coefficient of at least 0.999 was ensured during calibration.

### 4.3 Electrochemical Measurement (Rotating Ring Disk Electrode Analysis)

For electrochemical characterization, a glass cell, standard hydrogen reference electrode (RHE) from Gaskatel and glassy carbon rod (GCR 6/60 mm, redoxme) as counter electrode were used. To exclude contaminations RHE and CE were separated by porous glass frites. An image of the setup can be found in Figure S8 in the supporting information. 0.5 M H_3_PO_4_ served as the electrolyte, made from ultra‐pure water and 85 % o‐H_3_PO_4_ (EMSURE^®^, Merck). The RRDE (AFE7R9GCPT, Pine Research Instrumentation) included a glassy carbon disk with an area of 0.2475 cm^2^ equipped with a Pt‐ring of 0.1866 cm^2^ area and a collection efficiency of 37 %, given by the supplier. Before each test, the RRDE was polished with 1.00 μm and 0.05 μm aluminum oxide abrasion suspension (MicroPolish 40–10081, BUEHLER) for 5 min each followed by sonication in 2‐propanol and water for 5 min.

For the catalyst suspension 6 mg of catalyst were mixed with 561.6 μL H_2_O and 126 μL 2‐propanol and sonicated for 15 min in a sonication bath. Then, 76.2 μL of a 5 wt.% Nafion® solution in lower aliphatic alcohols were added, followed by 5 min bath sonication and 4 min of horn sonication (amplitude 10 %, 30 s on, 30 s off). The RRDEs were pre‐heated in oven at 60 °C, coated with 12.6 μL of ink and dried at 60 °C in oven for 5 min, resulting in a loading of 400 μg_Fe−N−C_ cm^−2^. The coated RRDEs were stored with a water droplet on the coated area until use to prevent contamination.

**Figure 8:**
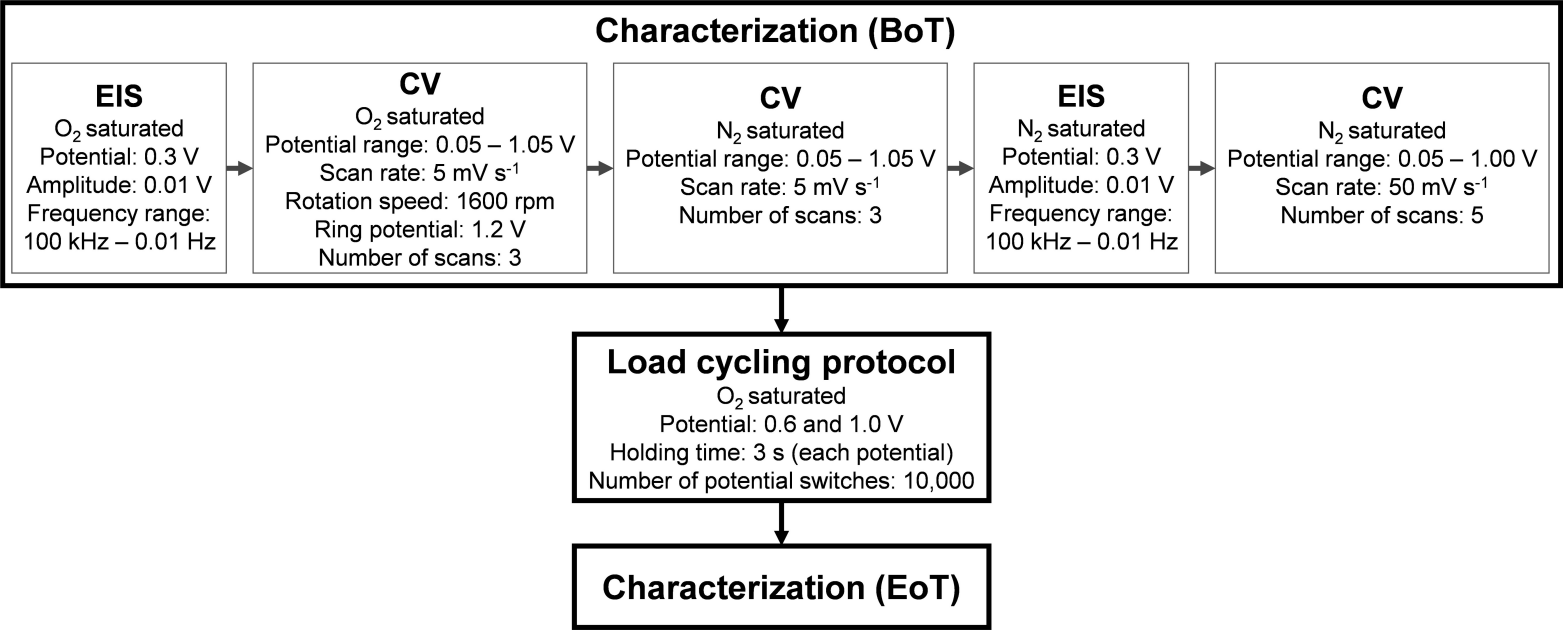
Diagram of measurement protocol for EIS, polarization curves, CVs and load cycling. The analysis was started with the begin of test (BoT) characterization, followed by the load cycling and the end of test (EoT) characterization, analog to the BoT. A minimum of 15 minutes of purging has been performed to saturated the electrolyte to with oxygen or nitrogen gas.

For determination of the ORR activity, selectivity and degradation behavior the catalysts were characterized according to Figure 8. All potentials were measured against a regular calibrated RHE and unless other stated the potential in this article is given in V vs. RHE.

The potentials are corrected by the internal resistance *R* which is extracted from EIS measurement. The i*R corrected potential is derived from *E*_*iR‐corr*_ =E_measured_ −(j_disk_*R), where *j_disk_* represents the background CV (3. Step in characterization in Figure 8) subtracted current density of the disk. The CVs, polarization and ring curves and of three individual measurement before the AST were averaged, and after the AST averaging of at least two curves were ensured, since some interference occurred during 17 h AST protocol. The *H_2_O_2_ yield* was calculated according to following formula, with a ring collection efficiency *N=*37 % given by the manufacturer.

(1) $$H_{2}O_{2}\;yield=\;\frac{200\frac{i_{ring}}{N}}{i_{disk}+\frac{i_{ring}}{N}}$$

First, the diffusion limited current density *j_lim_* is the mean value, extracted from the polarization curves in the diffusion limiting region in the area between 0.1 and 0.4 V. Then, the kinetic current density *j_kin_* is obtained by following formula.

(2) $$j_{kin}\;=\;\frac{j\;\cdot \;j_{lim}}{j_{lim}\;-j}$$

Afterwards, the MA was calculated by dividing *j_kin_* by the catalyst loading *m_catalyst_* (400 μg_Fe−N−C_ cm^−2^).

## Conflict of Interests

The authors declare no conflict of interest.

5

## Supporting information

As a service to our authors and readers, this journal provides supporting information supplied by the authors. Such materials are peer reviewed and may be re‐organized for online delivery, but are not copy‐edited or typeset. Technical support issues arising from supporting information (other than missing files) should be addressed to the authors.

Supporting Information

## Data Availability

The data that support the findings of this study are available from the corresponding author upon reasonable request.
