# Supplementary material for: Impact of Aerogel Modification for Fe−N−C Activity and Stability towards Oxygen Reduction Reaction in Phosphoric Acid Electrolyte
Source: ChemSusChem. 2024 Dec 19;18(8):e202401843. doi: 10.1002/cssc.202401843 (PMC11997938; doi:10.1002/cssc.202401843)
Supplement: Supplementary file 1 — Supporting Information [file CSSC-18-e202401843-s001.pdf]

ChemSusChem

Supporting Information

Impact of Aerogel Modification for Fe–N–C Activity and Stability towards Oxygen Reduction Reaction in Phosphoric Acid Electrolyte

Tanja Zierdt,* Torben Reuter, Julia Müller–Hülstede, Julia Buschermöhle, Dana Schonvogel, Jessica Kröner, Marina Schwan, Barbara Milow, Peter Wagner, and K. Andreas Friedrich

Supporting information

Impact of Aerogel Modification for Fe-N-C Activity and Stability towards Oxygen Reduction Reaction in Phosphoric Acid Electrolyte

Tanja Zierdt[a, b], Torben Reuter[c], Julia Müller-Hülstede[a], Julia Buschermöhle[a], Dana Schonvogel[a], Jessica Kröner[c], Marina Schwan[c], Barbara Milow[c], Peter Wagner[a], K. Andreas Friedrich[b, d]*

[a] T. Zierdt*, Dr. J. Müller-Hülstede, Julia Buschermöhle, Dr. H. Schmies, Dr. D. Schonvogel, P. Wagner
Institute of Engineering Thermodynamics
German Aerospace Center (DLR)
Carl-von-Ossietzky-Str. 15, 26129 Oldenburg (Germany)

[b] T. Zierdt*, Prof. Dr. K. Andreas Friedrich
Institute for Building Energetics, Thermotechnology and Energy Storage (IGTE)
University of Stuttgart
Pfaffenwaldring 31, 70569 Stuttgart (Germany)

[c] Torben Reuter, Jessica Kröner, Dr. Marina Schwan, Prof. Dr. Barbara Milow
Institute of Materials Research, Aerogels and Aerogel Composites
German Aerospace Center (DLR),
Linder Höhe, 51147, Cologne (Germany)

[d] Prof. Dr. K. Andreas Friedrich
Institute of Engineering Thermodynamics
German Aerospace Center (DLR)
Pfaffenwaldring 38-40, 70569 Stuttgart (Germany)

*E-mail: tanja.zierdt@dlr.de

Keywords: Fe-N-C, electrocatalyst, carbon aerogel, oxygen reduction reaction, phosphoric acid electrolyte

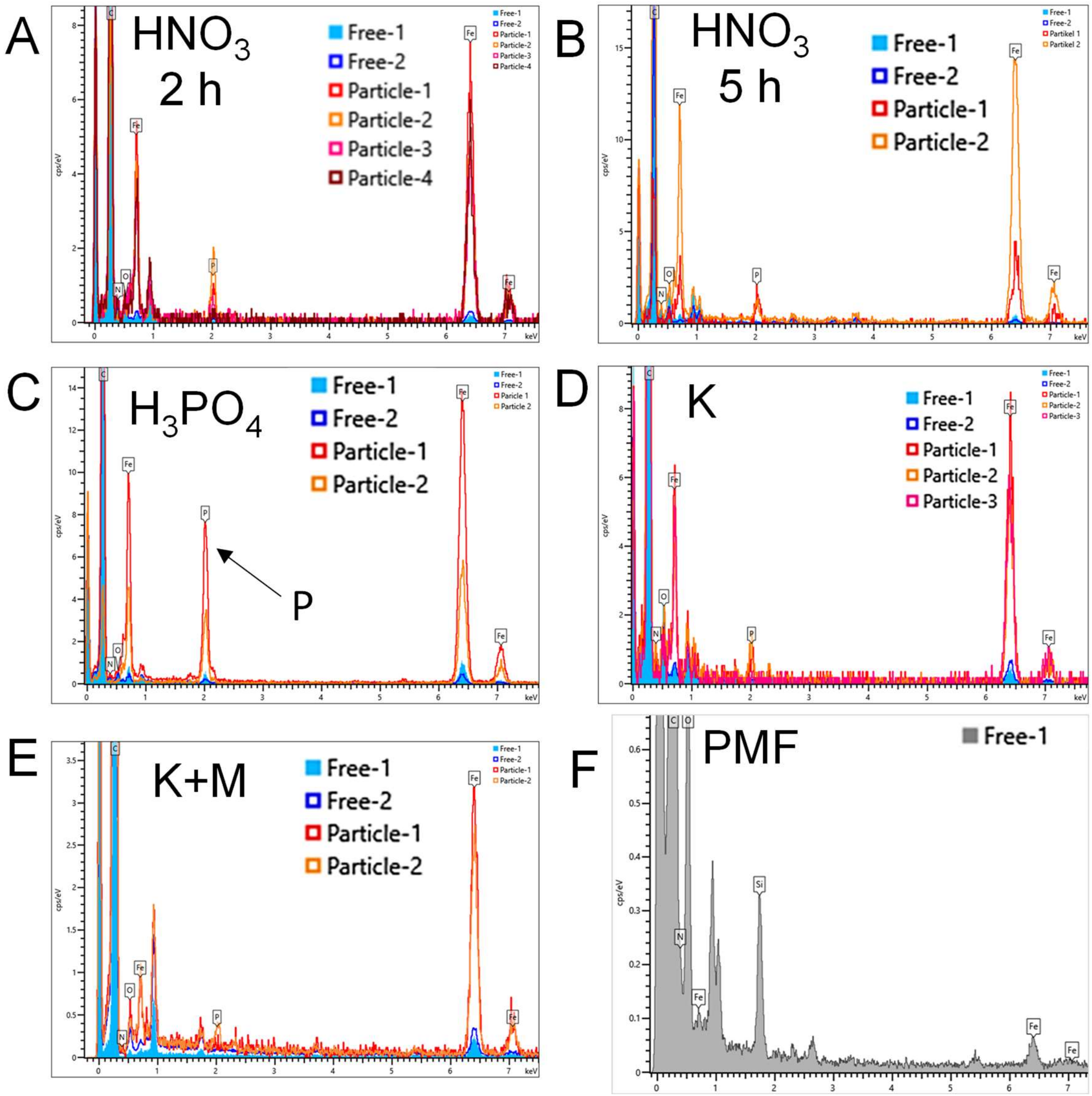

Figure S 1. Spectra of regions that are free of particles and TEM grid (denoted as "Free") in comparison to particles (denoted as "Particles"). For PMF no particles are observed.

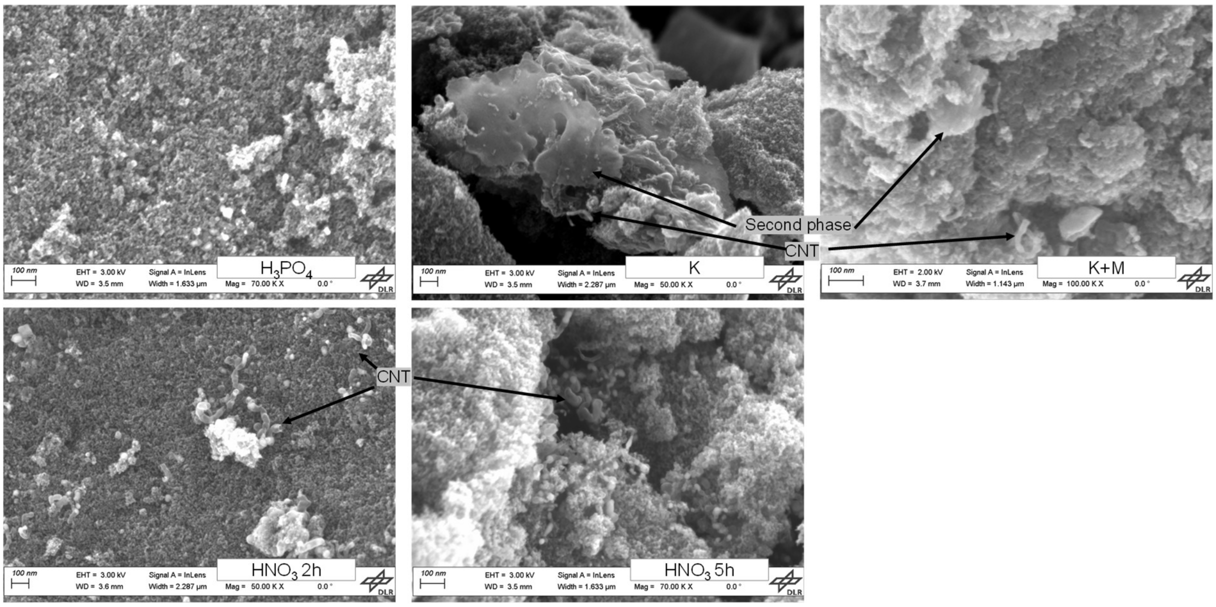

Figure S 2. Scanning electron microscopy images of Fe-N-C catalysts. K and K+M contain of a second, sheet-like structure in addition to the porous carbon. Carbon-nanotubes (CNT) are marked in the images.

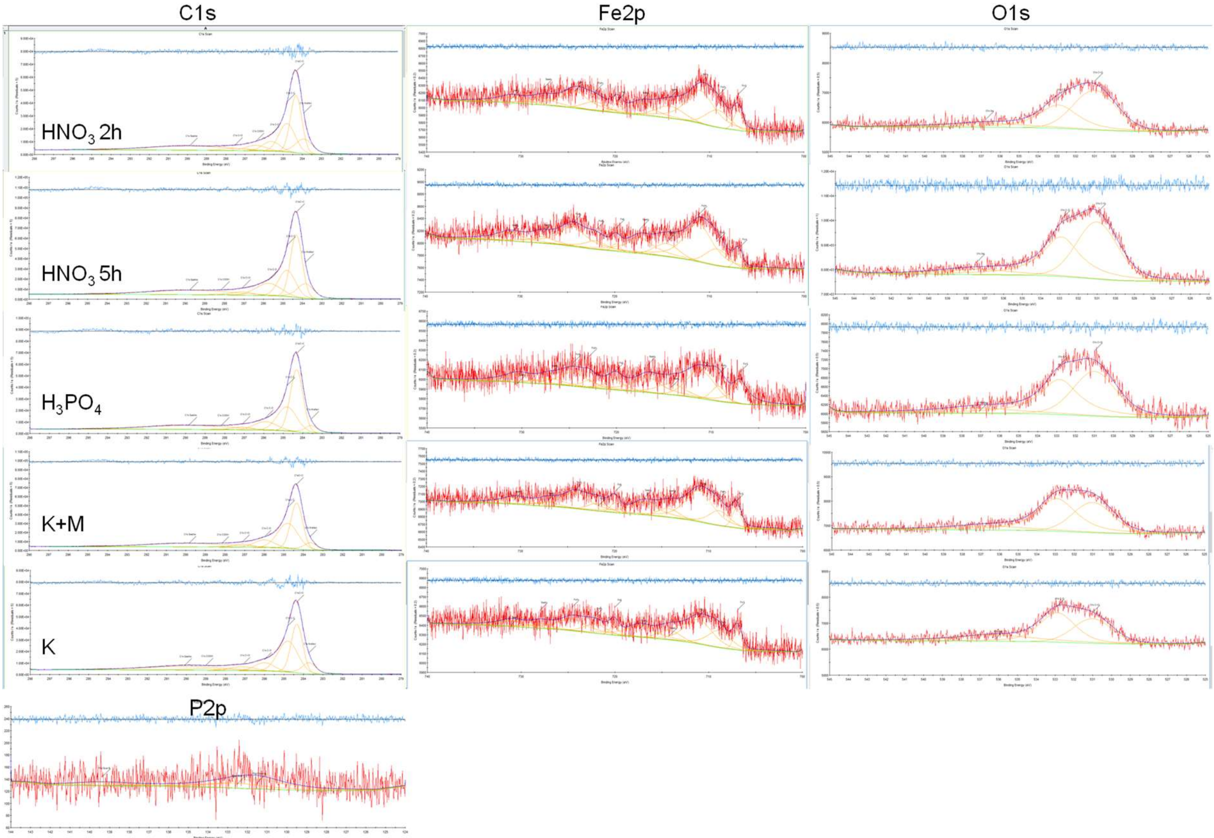

Figure S 3. XP spectra of the Fe-N-Cs. And P2p spectrum for Fe-N-C H_3PO_4.

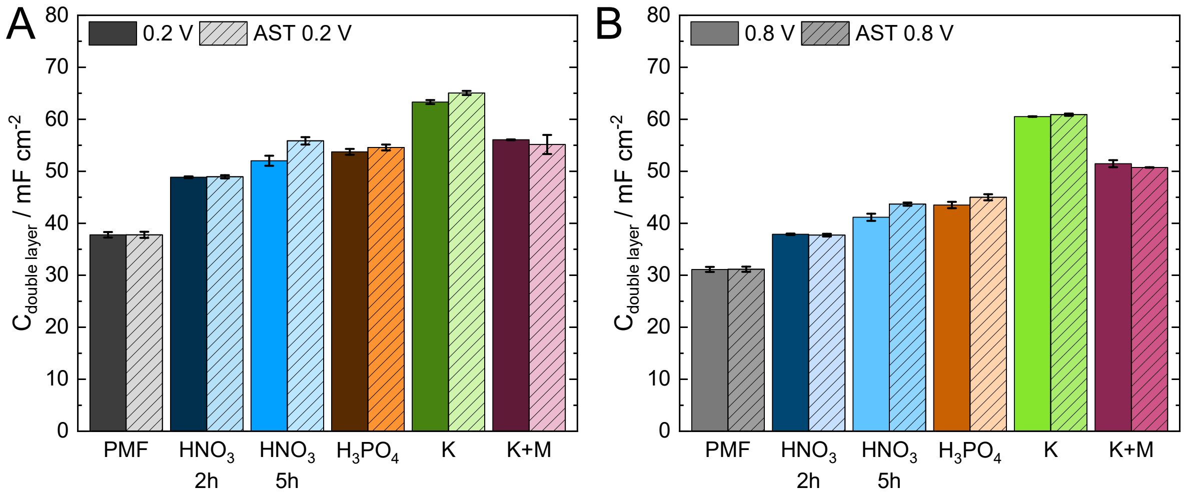

Figure S 4. Mean values of the double layer capacity of the cyclic voltammograms before and after the AST at 0.2 and 0.8 V.

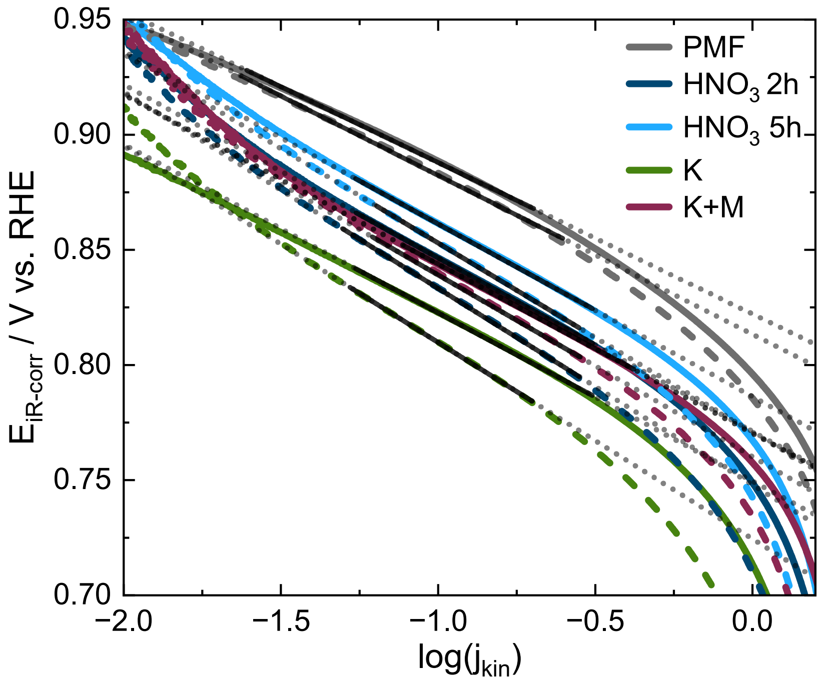

Fe-N-C:	PMF	HNO₃ 2h	HNO₃ 5h	K	K+M
**Before AST**	-62 ± 1	-75 ± 1	-75 ± 1	-73 ± 1	-74 ± 6
**After AST**	-65 ± 0	-85 ± 2	-82 ± 0	-85 ± 1	-80 ± 10

Figure S 5. Tafel plots and Tafel slopes of the Fe-N-Cs, with indication of the Tafel tangents in black. Comparison before (solid curves) and after (dashed curves) 10,000 potential square wave cycling AST between 0.6 and 1.0 V. Fe-N-C H_3PO_4 is excluded, as no reasonable Tafel plot is achievable.

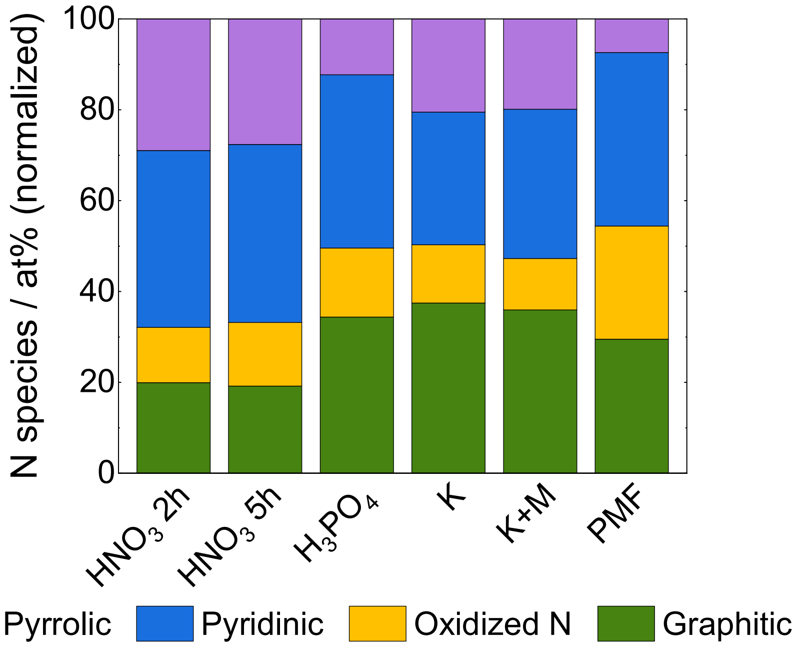

Figure S 6. N species content of the catalysts, normalized to 100 %.

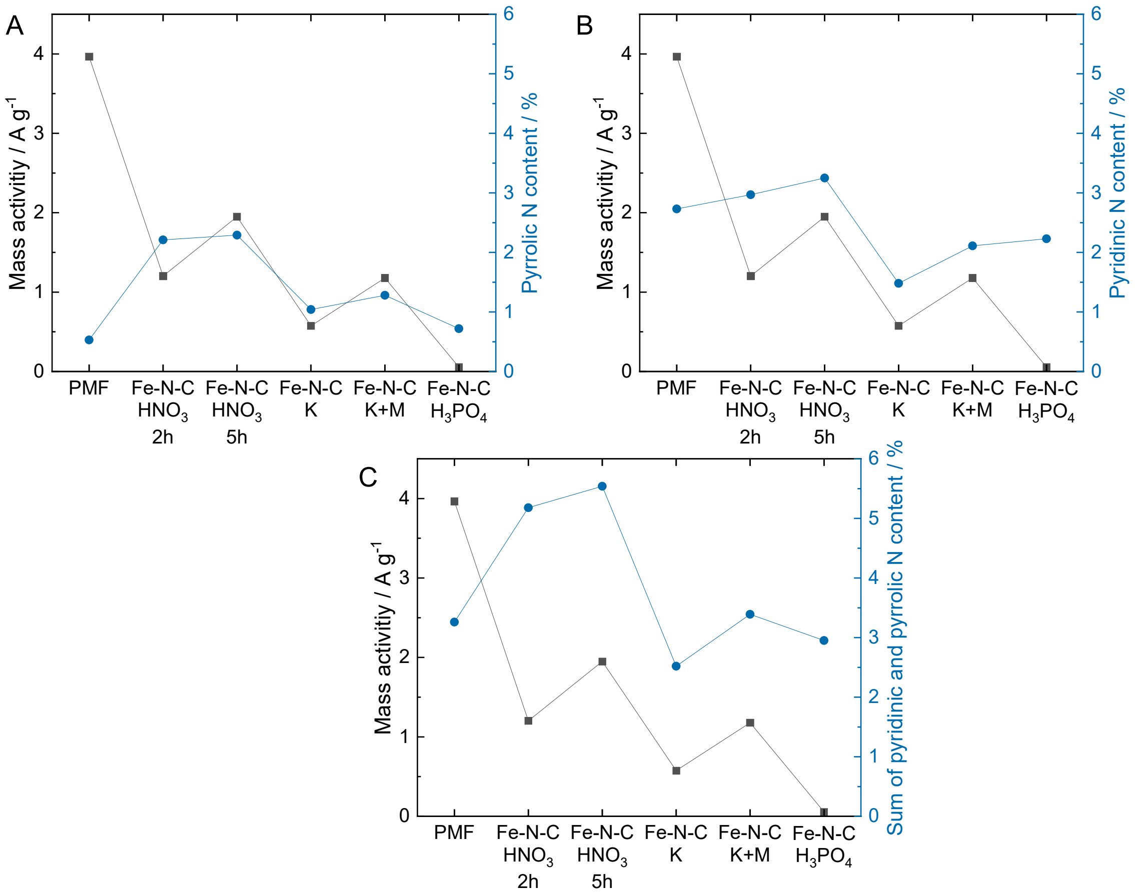

Figure S 7. Correlation between MA (represented by square points) on the left y-axis and the pyrrolic N content (A), the pyridinic N content (B) and the sum of pyridinic and pyrrolic N content (C) (represented by circle points) as a percentage of the total composition of the catalyst material on the right y-axis.

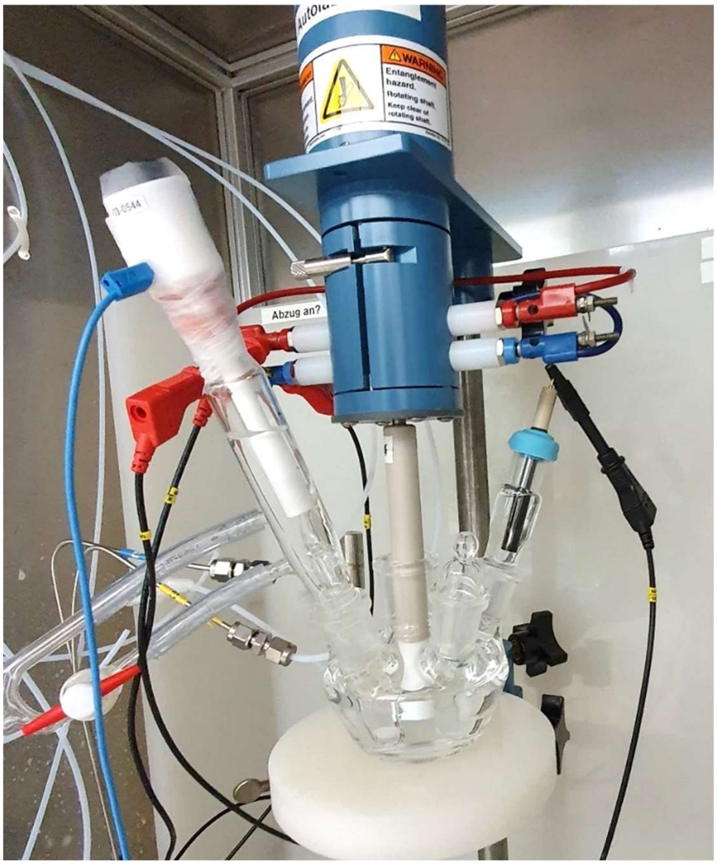

Figure S 8. Electrochemical RRDE setup.
